# Cancer du sein bilatéral synchrone: expériences du centre Mohammed VI pour le traitement des cancers CHU Ibn Rochd Casablanca

**DOI:** 10.11604/pamj.2016.25.121.9967

**Published:** 2016-10-27

**Authors:** Ahmadaye Ibrahim Khalil, Karima Bendahhou, Houriya Mestaghanmi, Rachid Saile, Abdellatif Benider

**Affiliations:** 1 Laboratoire de Physiopathologie et Génétique Moléculaire, Faculté des Sciences Ben M’Sik, Université Hassan II, Casablanca, Maroc; 2 Registre des Cancers de la Région du Grand Casablanca, Maroc; 3 Laboratoire de Biologie et Santé, Unité de Recherche Associée au CNRST-URAC 34, Faculté des Sciences Ben M’Sik, Université Hassan II, Casablanca, Maroc; 4 Centre Mohammed VI pour le Traitement des Cancers, CHU Ibn Rochd Casablanca, Maroc

**Keywords:** Cancer sein bilatéral, épidémiologie, clinique, histologie, thérapeutique, Bilateral breast cancer, epidemiology, clinical, histology, therapeutic

## Abstract

Les cancers du sein bilatéraux synchrones (CSBS) sont des maladies qui se caractérisent par une importante hétérogénéité clinique et morphologique avec une fréquence entre 1,5 et 3,2%. Les femmes traitées pour un cancer du sein unilatéral sont à haut risque de développer un cancer au niveau controlatéral. Le dépistage et les progrès de l’imagerie mammaire ont permis une augmentation de découverte des CSBS. L’objectif de notre travail est d’étudier les caractéristiques épidémiologiques, cliniques, histologiques, et thérapeutiques du cancer du sein bilatéral. Il s’agit d’une étude transversale étalée sur deux années des patientes prise en charge au centre Mohammed VI pour le traitement des cancers. L’analyse statistique des résultats a été réalisée par le logiciel R. 31 patientes ont présenté un CSBS, représentant 2,4% des cas du cancer du sein dans notre Centre. L’âge moyen était 47,8 ± 8,4 ans, 22,6% utilisaient des contraceptifs oraux. Une histoire familiale de cancer du sein était observée dans 22,6% des cas. Le type histologique le plus fréquent était le carcinome canalaire infiltrant (58,1%), Le grade SBR II et III étaient fréquents (38,7%). Les récepteurs hormonaux entaient positifs, aux progestérones (38,7%) et aux œstrogènes (41,9%). Le récepteur HER2 était surexprimé dans 20,0% des cas. 29,0% des cas ont bénéficié d’une hormonothérapie et 3,2% de thérapies ciblées. Notre étude a montré que le cancer du sein bilatéral représente une proportion faible, mais avec certaines particularités cliniques, différant du cancer du sein unilatéral.

## Abstract (en)

Synchronous bilateral breast cancers (SBBC) are characterized by extensive clinical and morphological heterogeneity, with an frequency between 1.5 and 3.2%. Women treated for unilateral breast cancer are at higher risk of developing contralateral breast cancer. Screening and advances in breast imaging have improved detection rates of SBBC. Our study aims to analyze the epidemiological, clinical, histological and therapeutic features of bilateral breast cancer. We conducted a cross-sectional study of patients with breast cancer treated at the Mohammed VI Center over a two year period. Statistical analysis of the results was performed using R. software. 31 patients had SBBC, representing 2.4% of breast cancer cases in our Center. The average age was 47.8 ± 8.4 years, 22.6% of patients used oral contraceptives. A family history of breast cancer was observed in 22.6% of cases. The most common histological type was invasive ductal carcinoma (58.1%), SBR grade II and III were common (38.7%). Hormone receptors were positive for progesterone (38.7%) and for estrogen (41.9%). HER2 was overexpressed in 20.0% of cases. 29.0% of patients received hormonal therapy and 3.2% targeted therapies. Our study showed that bilateral breast cancer represents a small percentage of all breast cancers but have specific clinical features that help to differentiate it from unilateral breast cancer.

## Introduction

Les cancers du sein bilatéraux synchrones (CSBS) ne sont pas exceptionnels et présentent une incidence entre 1,5 et 3,2% [1]. La première étude sur le cancer du sein bilatéral a été publiée par Kilgore et al. en 1921 [2], qui avaient définit le CSBS comme étant diagnostiqués simultanément. Haagensen et al. ont introduit la notion de délai entre le diagnostic des deux cancers [3]. La durée de ce délai a été définie arbitrairement par plusieurs auteurs, et varie d’un à six mois [4]. Les femmes traitées pour un cancer du sein unilatéral possèdent un haut risque de développer un cancer au niveau controlatéral. Ce risque est estimé 5 à 7 fois le risque de survenue d’un premier cancer primitif du sein dans la population générale [5]. La génération du dépistage systématique du cancer du sein par mammographie et les progrès de l’imagerie mammaire ont permis une large augmentation de la découverte des cancers du sein à un stade infra-clinique, mais aussi la prévalence des CSBS. La prise en charge thérapeutique des CSBS est largement discutée et elle variable selon les auteurs. Le geste chirurgical local est souvent radical (mastectomie) au détriment du traitement conservateur. De plus, les techniques d’évaluation de l’envahissement ganglionnaire axillaire sont en pleine évolution et l’intérêt de la technique du ganglion sentinelle dans cette indication n’a pas été évalué. La physiopathologie des CSBS peut être considérée actuellement comme une transformation maligne multiple avec des types histologiques et immuno-histochimiques semblables ou discordantes. L’objectif de cette étude est de préciser la fréquence, de définir les facteurs de risque, de déterminer les caractéristiques épidémiologiques, cliniques, histologiques et thérapeutiques et de montrer l’apport des différents types d’imagerie dans le diagnostic des CSBS.

## Méthodes

Nous avons étudié d’une manière rétrospective les patientes ayant un cancer du sein bilatéral prises en charges au sein du centre Mohammed VI pour les traitements des cancers, CHU Ibn Rochd, durant les deux années 2013-2014. Les patientes ont été sélectionnées sur certains critères d’inclusion (tumeurs malignes du sein diagnostiquées simultanément et jusqu’à six mois suivant le diagnostic de la première tumeur). Celles pour lesquelles une des tumeurs correspondait à une récidive d’un cancer ont été exclues. Les variables qui ont été colligées dans cette étude sont l’âge au diagnostic, la parité, le tabagisme, la ménopause, la prise ou non de contraceptif oral, l’antécédent familial du cancer du sein, la localisation de la tumeur, le stade clinique TNM, le type histologique, le grade histologique SBR, les récepteurs hormonaux et le Her2 et l’index de prolifération Ki-67. Nous avons également étudié la prise en charge thérapeutique des patientes et qui inclue l'administration d’une chimiothérapie néo adjuvante, le geste chirurgical réalisé (tumorectomie, mastectomie, geste axillaire) et les traitements adjuvants (chimiothérapie, radiothérapie, hormonothérapie). La collecte des variables était faite à l’aide d’une fiche standard et l’analyse des résultats grâce au logiciel R.

## Résultats

31 patientes présentant un cancer du sein bilatéral synchrone (CSBS) ont été prises en charge au sein de notre centre, avec une fréquence de 2,4% durant les deux années d’étude. L’âge moyen des patientes était de 47,8 ± 8,4 ans, avec des extrêmes (33-66 ans). 59,3% des cas étaient mariées au moment du diagnostic, la parité était de 2,5 ± 2,6 enfants, L’utilisation des contraceptifs oraux a été retrouvée dans 22,6% des cas (Tableau 1). La notion d’antécédent familial du cancer du sein était retrouvée dans 22,6% des cas. 85,7% des cas étaient du premier degré et 14,3% des cas étaient du deuxième degré. L’étude des circonstances diagnostiques des CSBS a montré que 70,4% des cas présentaient au moins un signe d’appel clinique: tumeur palpable, écoulement, mastite carcinomateuse, le diagnostic bilatéral clinique concernait 77,4% des cas. Dans 45,2% des cas, les tumeurs étaient situées dans le même quadrant dans chacun des deux seins. Chez 19,4% des patientes, la localisation tumorale était dans le quadrant supéro-externe. La taille de la tumeur du premier cancer variait entre un et six centimètres avec une moyenne de 3,2 cm. Les tumeurs étaient classées T1 dans 48,4%, T2 dans 25,8%, T3 dans 6,5% et T4 dans 19,4%. L’atteinte des ganglions était observée dans 25,9% des cas. Sur le plan histologique, dans tous les cas le deuxième cancer était identique au premier. Il s’agissait de 58,1% des cas de carcinome canalaire infiltrant, 9,7% de carcinome médullaire infiltrant, 9,7% de carcinome lobulaire infiltrant (Tableau 2). Le grade histologique de Scarff Bloom et Richardson (SBR) était observé dans le grade I (22,6% des cas) et dans le grade II et III (38,7% des cas) pour chaque grade. Une positivité des récepteurs hormonaux était remarquée, aux œstrogènes (41,9%) et à la progestérone (38,7%). Le Her2 était positif dans 20,0% des cas (Tableau 2). Les modalités thérapeutiques du premier cancer étaient effectuées dans 87,1% des cas par l’association de la chirurgie, la chimiothérapie et la radiothérapie. Toutes les patientes avaient subi un geste chirurgical, la mastectomie bilatérale dans 67,9% des cas et 32,3% une mastectomie unilatérale et 67,7% des cas tumorectomie bilatérale. Dans notre série, aucune patiente n’avait bénéficié d’un geste de reconstruction mammaire. La radiothérapie postopératoire était pratiquée dans 38,7% des cas, la chimiothérapie adjuvante dans 51,6% des cas, l’hormonothérapie à base de Tamoxiféne était préconisée dans 29,0% des cas et la thérapie ciblée à base de l’Herceptine dans 3,2% des cas. Sur le plan pronostique, 19,4% ont présenté des métastases dans un délai de 1 à 9 mois après le traitement du deuxième cancer. Parmi ces patientes, 96,8% ont été suivies avec un recul de 3 à 24 mois. Un seul cas a subi une rechute et un seul cas était perdu de vue.

**Tableau 1:** caractéristiques épidémiologiques des patientes traitées pour un CSBS

	Moyen (écart type)	Extrêmes
Age (années)	47,8 ± 8,4	33-66
Age de la puberté	13,4 ± 1,6	10-17
Age a la ménopause	49,6 ± 7,0	35-58
Age a la première grossesse	23,1 ± 5,3	31-19
Parité	2,5 ± 2,6	0-111
	**Nombre (N=31)**	**Pourcentage (%)**
Utilisation des contraceptifs oraux	7	22,6
Ménopause	8	25,8
Antécédent familial du cancer du sein	7	22,6
1^er^ degré	6	85,7
2^eme^ degré	1	14,3

**Tableau 2:** différentes type histologiques des CSBS

	Effectif N=31	Pourcentage (%)
Carcinome invasif	3	9,7
Carcinome médullaire	3	9,7
Carcinome mucineux	1	3,2
CCI	18	58,1
CLI	3	9,7
Autres	3	9,7

(CCI: carcinome canalaire infiltrant, CLI : carcinome lobulaire infiltrant)

## Discussion

Le cancer du sein bilatéral est un cancer malin, atteignant les deux seins simultanément ou à des moments différents [6]. La bilatéralité ainsi que la multicentricité d’un cancer reflète en réalité la capacité de la transformation maligne, d’apparaître à des endroits différents du tissu mammaire de façon indépendante, simultanée ou non [7]. Cette différence est certainement liée à un certain nombre de facteurs de risque. Plusieurs critères permettent de distinguer un réel deuxième cancer primitif du sein, d’une localisation métastatique à ce niveau [8]. Cependant, les deux cancers sont de types histologiques différents; la présence d’une composante in-situ dans le cancer du sein controlatéral; le degré de différenciation du deuxième cancer est plus élevé par rapport au premier cancer; l’absence de métastase lors du traitement du premier cancer avec un intervalle entre les deux cancers de plusieurs années [9]. Selon la littérature, le délai distinguant les cancers du sein bilatéraux synchrone des cancers du sein métachrones varie de six à douze mois. Pour certains auteurs, le cancer du sein controlatéral est synchrone s’il est découvert pendant le traitement du premier cancer ou dans le mois qui suit [10], alors que pour d’autres auteurs, dans les six mois qui suivent [9], ou les douze mois après le traitement du premier cancer [10, 11]. Dans notre série, la fréquence du CSBS est de 2,4%. Ce résultat concorde avec les taux d’incidence rapportés dans la littérature, qui varient de 0,8 à 3,2% [6], alors qu’elle est faible par rapport à celle rapportée par Houssine et al. (3,5%) [12]. Par ailleurs, plusieurs auteurs ont montré que le risque d’apparition d’un deuxième cancer primitif au niveau du sein controlatéral est estimé entre 5 et 7 pour 1000 femmes par années [4, 13, 14].

Plusieurs études rapportent les caractéristiques épidémiologiques des patientes présentant un CSBS. L’âge plus jeune et le statut pré-ménopausique sont des facteurs de risque parfois décrit dans la littérature [11]. Dans notre série, l’âge moyen des patientes était de 47,8 ans, celui-ci reste peu élevé par rapport à celui observé par Houssine et al. [12]. Par contre, Lebris et al. ont montré un âge moyen de 60,1 ans [13]. D’autres études comparant le CSBS aux cancers du sein unilatéraux, montrent que l’âge jeune et le statut pré-ménopausique ne sont pas considérés comme des facteurs de risque du cancer du sein bilatéral [15]. Le risque d’apparition d’un cancer du sein controlatéral est marqué quand l’âge de la femme est inférieur à 40 ans au moment du traitement du premier cancer, avec un risque de 5 à 10 pour 1000 femmes par années [16, 17].

En revanche, ce risque est plus faible si le premier cancer est traité à un âge post-ménopausique ou s’il est inférieur à 4 pour 1000 femmes par année [17]. La présence des antécédents familiaux du cancer du sein est un facteur de risque important d’apparition d’un cancer du sein controlatéral. Dans notre série, 22,6% des patientes avaient au moins un antécédent familial du cancer du sein. 85,7% des cas étaient du premier degré et 14,3% du deuxième degré. Ce chiffre concorde à ceux retrouvés dans la littérature. En effet, De La Rochefordiere et al. [16] ont montré dans leur série que 23,0% des cas avaient au moins un antécédent familial du cancer du sein. Il en est de même pour les travaux de Newman et al. qui ont observé 23,7% des cas ayant au moins un antécédent familial [18]. Par contre, Lebis. et al. [13] ont relevé 41,3% des cas. Plusieurs auteurs considèrent la présence d’antécédents familiaux comme un facteur de risque de CSBS [19]. Cependant, l’importance de ce facteur est discutée dans les travaux de Newman et al. [18] et Jobasen et al. [20], qui ne distinguent pas entre cancers synchrones et métachrones et qui ont remarqué aussi que les antécédents familiaux ne sont pas plus fréquents chez les femmes ayant un cancer du sein bilatéral que celles ayant un cancer du sein unilatéral [20]. Par ailleurs, Kwast et al. ont montré que le risque de cancer du sein controlatéral était augmenté chez les femmes ayant un antécédent familial au premier degré (risque relatif -1,91, IC 95% (1,22-2,99) [21].

Dans notre série, nous n’avons pas effectué d’étude sur les mutations BRCA1 et BRCA2 chez les patientes présentant un CSBS. Dans la littérature, il n’existe pas de travaux traitant spécifiquement l’incidence de ces mutations chez les patientes ayant un CSBS. Chez les patientes ayant une mutation BRCA1 avec un premier cancer du sein, le risque du cancer controlatéral est évalué à 48% à l’âge de 50 ans et à 64% à l’âge de 70 ans [22]. Le seul facteur de risque épidémiologique retrouvé de CSBS est l’antécédent personnel du cancer du sein, avec un taux d’incidence annuel du cancer controlatéral et qui est évalué à 0,5-0,8% [6]. Selon les données de la littérature, il n’existe pas des données concernant l’antécédent personnel et familial des cancers non mammaires. Kwast et al. ont montré que les patientes ayant un antécédent de cancer du sein bilatéral ont un risque augmenté du cancer par rapport aux patientes ayant un cancer mammaire unilatéral (cancer de l’ovaire, de l’endomètre, ORL, cutané, colorectal, pulmonaire et hématologique [21]. Ceux-ci pourraient être, d’une part en rapport avec des facteurs génétiques et d’autre part, des facteurs environnementaux, notamment les effets secondaires du traitement (chimiothérapie, hormonothérapie et radiothérapie) du cancer du sein [21]. L’analyse des circonstances diagnostiques des CSBS met en évidence un taux relativement important des cancers infra-cliniques (65,2%) [13]. Dans notre série, 96,8% des tumeurs controlatérales sont relevées par imagerie conventionnelle. L’examen clinique quant à lui n’a permis le diagnostic des tumeurs controlatérales que chez 13% des cas. Dans la littérature, les taux retrouvés des cancers sans point d’appel clinique sont inférieurs à ceux observés dans notre série. Par exemple, dans la série de Polednak (1984-1999) [14], les cancers bilatéraux qui n’avaient pas de traduction clinique étaient observés dans 34,7% des cas. Alors que Marpeau et al. (1992-2006) ont observé un taux de 26,8% des cas [4], et Hungness et al. (1984-1999) [23], 19,0% des cas. Par contre De la Rochefordiere et al. (1984 1999) n’ont observés que 7% des cas [16]. Ces différences pourraient être dues à la génération du dépistage mammographique du cancer du sein permettant un diagnostic plus précoce de lésions non palpables.

L’étude histologique des tumeurs dans le cadre des CSBS est un élément important. Dans notre série, le carcinome canalaire infiltrant a été retrouvé dans 64,6% des cas alors que le carcinome lobulaire infiltrant dans 9,7% des cas. La deuxième tumeur du sein était de même type histologique que le premier dans tous les cas observés. Le caractère multifocal ou multicentrique du premier cancer primitif du sein est fortement lié à un risque de bilatéralité [24]. Par ailleurs, certains auteurs rapportent une forte proportion de bilatéralité chez les patientes ayant un cancer multicentrique et de type histologique in situ [25]. L’étude des grades histologiques des tumeurs bilatérales selon la classification de Scarff Bloom et Richardson a montré que 38,7% des cas sont de grade I, 22,6% des cas de grade II et 38,7% des cas de grade III. Selon la littérature il n’existe pas de données qui considèrent le grade élevé d’une tumeur comme un facteur de risque de cancer controlatérale. Lesser et al. n’ont pas observé de corrélation significative entre la bilatéralité et le grade histologique [24]. Dans notre série, le siège du premier cancer primitif était observé dans 19,4% des cas au niveau du quadrant supéro-externe, 3,2% des cas au niveau du prolongement axillaire et 3,2% des cas avaient une localisation rétro-mamelonnaire. Webber et al. ont retrouvé fréquemment une récidive controlatérale chez les patientes qui avaient une localisation rétro-mamelonnaire [26].

Dans notre série, 41,9% des cas exprimaient des récepteurs aux ostéogènes et 38,7% des cas des récepteurs à la progestérone. Selon la littérature, Intra et al. [25] de même Matsuo et al. [27] ont montré un taux d’expression des récepteurs hormonaux significativement plus élevés chez les patientes avec un CSBS. Alors que pour d’autres auteurs, les CSBS auraient une expression plus forte des récepteurs aux œstrogènes [28]. Cette hormono-sensibilité globalement augmentée pourrait suggérer un potentiel d’agressivité biologique moindre. L’atteinte ganglionnaire rapportée dans notre série était de 25,9% des cas. Des résultats similaires ont été observés par d’autres auteurs, De La Rochefordiére et al. Hungness et al. ainsi que Heron et al. et Intra et al. qui ont rapporté des taux respectifs de 26%; 32%; 37,2% et 47,4%. [16, 24, 26, 29]. Le récepteur HER2 a été surexprimé dans 20,0% des cas. Cette surexpression de Her2 dépasse celle de la littérature. D’ailleurs, Marpeau et al. et Intra et al. ont observé respectivement la forte surexpression de Her2 dans 9,1% et 13,3% des cas [4, 25]. Dans notre étude, en ce qui concerne le stade au moment du diagnostic, nous avons observé que 38,7% des cas présentaient le stade I, 19,4% des cas le stade II, 22,6% des cas le stade III et 19,4% des cas le stade IV. La plupart des études ont montré que la survenue d’un deuxième cancer primitif du sein opposé n’altère pas grandement le pronostic, à condition qu’il soit traité convenablement en fonction du stade lors du diagnostic et de l’état des ganglions. En cas de présence de facteurs de haut risque de bilatéralité (âge très jeune, antécédent familiaux de cancer du sein ou cancer multifocal), aucun geste chirurgicale sur le sein controlatéral n’est indiqué en l’absence de lésion suspecte à l’examen clinique ou à la mammographie [18].

Dans notre série, les modalités de traitement étaient une mastectomie unilatérale du sein primitif dans 67,7% des cas, la chimiothérapie adjuvante dans 51,6% des cas, la radiothérapie était pratiquée dans 38,7% des cas et l’hormonothérapie dans 29% des cas. Il est à noter aussi que pour les patientes qui procèdent régulièrement à des examens physiques et mammographiques, le deuxième cancer du sein controlatéral sera découvert à un stade plus précoce, permettant ainsi un traitement aussi efficace que le premier cancer [6]. En outre, la survenue d’un deuxième cancer du sein controlatéral n’est pas forcément une contre-indication au traitement conservateur. Dans la série d’Egan, 87% des cas traités de manière conservatrice lors du premier cancer, ont bénéficiés du traitement conservateur pour leur deuxième cancer [29]. Gollamudi et al. ont montré que les patientes ayant un CSBS diagnostiqué à un stade I ou II, ont bénéficié d’un traitement conservateur pour les deux seins et rapportent la comparaison avec les patientes ayant un cancer du sein unilatéral, un même taux de survie à cinq ans a été observé, ainsi qu’un même résultat esthétique [30]. Selon la littérature, les données concernant le pronostic et l’impact de la survenue du deuxième cancer sont divergentes. Si le suivi de notre population est limité pour en apprécier la survie, celle-ci a été étudiée par plusieurs auteurs. Polednak a montré que le taux de survie global à cinq ans varie entre 65 à 86% [14]. Mais la majorité des auteurs rapportent un taux supérieur à 80%. Le pronostic des CSBS dépend principalement du stade au moment du diagnostic de la tumeur.

## Conclusion

Le cancer du sein bilatéral synchrone ne représente pas une entité exceptionnelle. Si le principal facteur de risque de la survenue d’un cancer du sein controlatéral est l’antécédent personnel du cancer du sein, la notion d’antécédent familial du cancer du sein a été relevé dans 22,6% des cas; la présence d’un carcinome lobulaire infiltrant et la présence de tumeur multifocale permettent de guider vers une exploration plus précise du sein controlatéral. La génération du dépistage systématique du cancer du sein par mammographie et les progrès de l’imagerie mammaire ont permis une augmentation de la découverte de cancers du sein à un stade infra-clinique, mais aussi de la prévalence des cancers du sein bilatéraux synchrones.

### Etat des connaissances actuelles sur le sujet

- Les cancers du sein bilatéraux synchrones (CSBS) sont des maladies qui se caractérisent par une importante hétérogénéité clinique et morphologique avec une fréquence entre 1,5 et 3,2% ;
- Le délai distinguant les cancers du sein bilatéraux synchrone des cancers du sein métachrones varie de six à douze mois.

### Contribution de notre étude à la connaissance

- C’est un travail réalisé la pour la première fois au centre Mohammed VI pour le traitement des cancers l’un des deux plus grand centre pour la prise en charge et le traitement des cancers au Maroc;
- La fréquence du cancer du sein bilatéral synchrone au sein de notre centre;
- Etude des facteurs de risque, les caractéristiques épidémiologiques, cliniques, histologiques et thérapeutiques et l’apport des différents types d’imagerie dans le diagnostic des CSBS.
